# Feeding Strategies of Brown Howler Monkeys in Response to Variations in Food Availability

**DOI:** 10.1371/journal.pone.0145819

**Published:** 2016-02-05

**Authors:** Óscar M. Chaves, Júlio César Bicca-Marques

**Affiliations:** Pontifícia Universidade Católica do Rio Grande do Sul, Faculdade de Biociências, Porto Alegre, Rio Grande do Sul, Brazil; NYIT College of Osteopathic Medicine, UNITED STATES

## Abstract

Primates display varying degrees of behavioral flexibility that allow them to adjust their diet to temporal changes in food availability. This trait might be critical for the survival of folivorous-frugivorous species inhabiting small forest fragments, where the availability of food resources tends to be lower than in large fragments and continuous forests. However, the scarcity of studies addressing this issue hampers our understanding of the adaptive behaviors that favor the survival of these primates in low-quality habitats. We conducted a 36-mo study testing the hypothesis that brown howler monkeys (*Alouatta guariba clamitans*) are able to adjust their diet in response to local and seasonal changes in resource availability. We compared the diet of six free-ranging groups inhabiting three small (<10 ha) and three large (>90 ha) Atlantic forest fragments in southern Brazil and estimated the temporal availability of their top food species (*i*.*e*., those species that together contribute ≥80% of total feeding records). We found that brown howlers exploited similarly rich diets in small (45, 54, and 57 plant species) and large (48, 51, and 56 species) fragments. However, intermonth diet similarity was higher for groups in small fragments, where howlers also fed on plant items from nine alien species. Fruits and leaves were the most consumed plant items in both small (42% and 49% of feeding records, respectively) and large (51% and 41%, respectively) fragments. The consumption of young leaves was higher in small than in large fragments, whereas the consumption of other plant items did not show a pattern related to fragment size. Regarding the contribution of growth forms as food sources, only the exploitation of palms showed a pattern related to fragment size. Palms contributed more to the diet of groups inhabiting large fragments. The availability of seasonal food items–ripe fruits and young leaves–influenced their consumption in both habitat types. Therefore, brown howlers cope with local and seasonal fluctuations in food availability by opportunistically exploiting resources. We believe that this feeding flexibility is a key component of the phenotypic plasticity that enables howlers to thrive in disturbed habitat patches, where periods of scarcity of preferred foods shall be more common.

## Introduction

The ability of an organism to survive in a changing and unpredictable environment is strongly related to its phenotypic plasticity. Phenotypic plasticity is the ability of a single genotype to manifest a range of phenotypes in response to variations in the environment [1, 2]. This ability is often considered adaptive because it may increase its bearer’s fitness [1–3]. Plasticity encompasses the flexibility in morphological traits, behavior, life history, physiology, and biochemistry among virtually all other traits [1, 2]. Behavioral flexibility is a crucial component of phenotypic plasticity. It allows individuals to change their behavior (both qualitatively and quantitatively) in space and time in response to environmental pressures [1, 2, 4]. It has been reported in insects [2], fishes [5], birds [6], bats [7], and primates [4, 8].

Behavioral flexibility is particularly relevant in the context of anthropogenic change due to its potential implications in conservation biology [2–4]. Human disturbances such as deforestation, forest fragmentation, and hunting, may severely reduce the amount and quality (*e*.*g*., low availability of food resources) of available habitat [9–11]. Under these circumstances, the short- and long-term survival of animal populations may depend more heavily on the ability of individuals to employ adaptive behavioral adjustments to the new condition than on their dispersal ability or the evolution via genetic changes [3, 12], as has been proposed for explaining the success of species surviving in fragmented and/or urban habitats [4, 6, 13, 14]. This research area should be a priority for conservation biologists because of the high contemporary extinction rates reported for most vertebrate groups [15].

Tropical primates are among the most affected animals by human disturbance of forests because they often depend on resources provided by large trees [16–19]. Therefore, it is expected that primates living in human-modified habitats and seasonal forests present a high behavioral flexibility to deal with shifts in resource availability [4, 20, 21]. The ability to vary diet composition by tracking seasonal changes in food availability is well known in New World (*e*.*g*., *Alouatta palliata*, *Ateles geoffroyi*, and *Cebus capucinus* [22]; *Sapajus apella* and *Saguinus* spp. [23]) and Old World (*e*.*g*., *Cercopithecus* spp. and *Colobus badius* [24]) monkeys. Consequently, many primates show high variations in the amount of fruit and leaves eaten along the year [4, 21, 22, 25].

Primates face additional pressures that favor behavioral flexiblity in human-modified habitats. For instance, within-species comparisons support the contention that individuals belonging to populations inhabiting small or disturbed fragments show more variable feeding behaviors than those living in large fragments (*e*.*g*., *Ateles geoffroyi* [26], *Alouatta* spp. [20, 27]). Primates can increase the consumption of fallback foods (*i*.*e*., food items from non-preferred species exploited when items from preferred ones are scarce [28]), low-digestible resources, such as leaves [27, 29, 30], and plant items from vines, lianas and palms, common growth forms in disturbed habitats [26, 30, 31]. They can also supplement the diet with items from alien plant species found in the anthropogenic matrix [32–34].

Howler monkeys (*Alouatta* spp.) are Neotropical primates well known for their behavioral flexibility, particularly their ability to cope with the scarcity of preferred foods in small and disturbed forest patches [20, 29, 35]. However, there is a bias in the howler literature towards the Mesoamerican *A*. *palliata* and *A*. *pigra* (*e*.*g*., [35–37]) and, to a lower degree, the South American *A*. *caraya* [32, 38, 39]. Publications on other South American taxa, such as the brown howler monkey (*A*. *guariba clamitans*), are scarce [40]. Moreover, all published and unpublished studies conducted so far on the later taxon are short-term (≤12 months), and only a few of them addressed how the temporal variation in food availability influences its feeding behavior (reviewed by [20, 40]).

This taxonomic bias and focus on short-term studies compromise our ability to understand the responses of *Alouatta* spp. to spatial and seasonal resource scarcity. In this respect, the brown howler monkey is a good model to assess dietary adjustments in response to shifts in food availability in large and small fragments. It inhabits a variety of forest fragments in the Atlantic forest [41], occurs at the southern limit of primate distribution in the Americas [42], where climatic seasonality is more pronounced, and exploits a diet composed of a mixture of items from native and alien plant species [40].

We conducted a 36-mo study on six social groups inhabiting three small and three large forest fragments to test the hypothesis that brown howlers can adjust their diet in response to local and seasonal changes in food availability. All study groups inhabited forests expected to have similar species composition by belonging to the same original physiognomic type within the same region to minimize the potential effect of differing plant assemblages on diet composition, an effect that has rarely been addressed (reviewed by [20, 40]). Specifically, we evaluated the following aspects of their diet at the group level: overall species richness, contribution of alien species, top food species, main plant items and growth forms, and the relationship between seasonal resource availability and consumption. Considering that food availability correlates with fragment size [35] and that howlers rely on both native and alien plant species in fragmented and disturbed habitats [29, 32], we expected that brown howlers:

1. exploit similarly-rich diets in both small and large fragments as indicated by a recent comprehensive review [40];
2. exploit more alien species in small than in large fragments because the first are more likely to be found near human settlements and contain planted fruiting species and because the anthropogenic matrix is important for primate feeding supplementation [43, 44];
3. show a higher intermonth diet similarity in small than in large fragments because the potentially poorer plant assemblages in the former [18, 35] might force animals to exploit food items from a lower number of species;
4. spend more feeding records to leaves and less records to fruit in small than in large fragments because fruit availability tends to be lower in the former (*e*.*g*., *Alouatta* spp. [20, 35], *Ateles geoffroyi* [26]);
5. spend more feeding records to plant items from non-tree growth forms such as palms, lianas, and vines, in small than in large fragments because of the presumably higher availability of these growth forms in the former (*e*.*g*., lianas [31, 45]); and
6. feed on plant items from native top food species, particularly the highly seasonal ones (ripe fruit, flowers [46], and young leaves [47]), according to their availability (*e*.*g*., *Alouatta pigra* [48]).

## Materials and Methods

### Ethics Statement

This study was approved by the Scientific Committee of the Faculty of Biosciences of the Pontifical Catholic University of Rio Grande do Sul (project #3477-SIPESQ). It meets all Brazilian animal care policies (permits #28578-SISBIO/ICMBio and #372-SEMA) and all ethical and legal requirements established by the American Society of Primatologists, Animal Care and Use Committee, and the Ethical Committee of the Zoological Society of London for research with nonhuman primates.

### Study Sites and Groups

**Fig 1:**
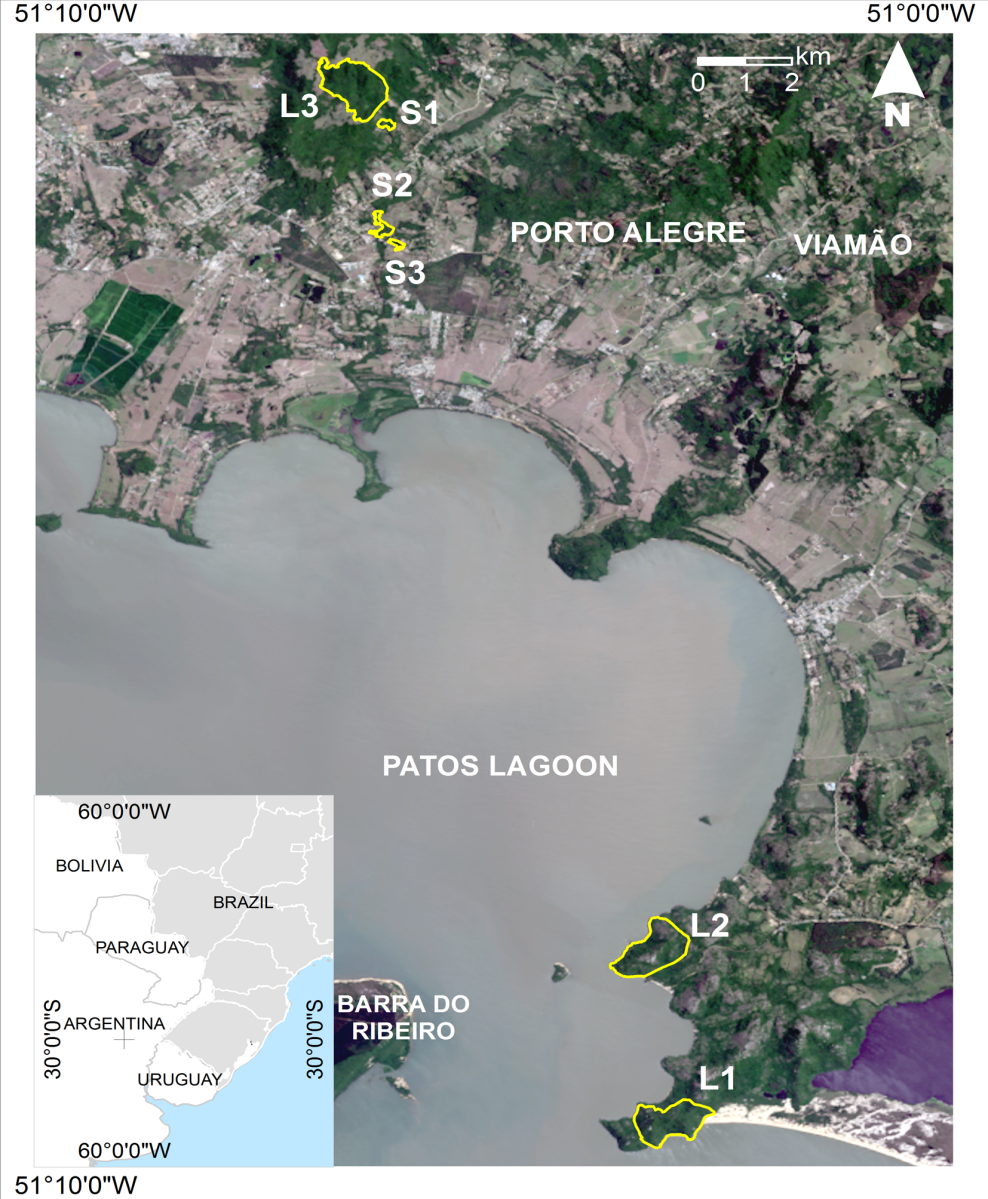
Location of study sites in southern Brazil. Study fragments delimited in yellow. Lansat8 open-access image (available at http://earthexplorer.usgs.gov/).

This study was conducted in three small (<10 ha: S1, S2, and S3) and three large (>90 ha: L1, L2, and L3) Atlantic forest fragments in Rio Grande do Sul State, southern Brazil (Fig 1). The distance between fragments ranged from 0.4 km (S2 and S3) to 23 km (L1 and L3; Fig 1). The three large fragments were within legally protected areas: L1 (93 ha) and L2 (106 ha) in the State Park of Itapuã, and L3 (108 ha) in the São Pedro Wildlife Refuge. On the other hand, only S1 (1.6 ha) is inside an unofficially protected area (the Econsciência private reserve), whereas S2 (9.5 ha) and S3 (2.9 ha) are unprotected fragments surrounded by human settlements, pastures, and cultivated lands (Fig 1).

Subtropical semideciduous forest covered all study sites [49]. Tree surveys (see below) showed that *Sebastiania serrata*, *Guapira opposita*, *Myrsine umbellata*, and *Casearia sylvestris* dominate the forests of the study fragments. These four dominant species are common Atlantic forest trees in Rio Grande do Sul State [50]. The importance value indices (IVI) of all tree species, and the sum of IVI and tree density for the top food species in each fragment are shown in S1 Table. The region is characterized by four clearly distinct seasons. According to our meteorological records, the average monthly temperature during the study period was 22°C. The highest monthly temperatures were recorded during the Summer (22°C-34°C), whereas the lowest occurred in the Winter (7°C-26°C). Annual rainfall was 1,187 mm (2012) and 1,071 mm (2013). Although it rains throughout the year, precipitation was higher during the Winter (especially in July and August).

We studied one social group per fragment. Their sizes ranged from 6 to 10 individuals (S2 Table). The groups inhabiting small fragments were habituated to people prior to the study because their home ranges were near human settlements. The habituation process of L1 and L3 study groups lasted from March to June 2011, whereas the habituation of L2 lasted from July to October 2011. Whereas two groups inhabited S2, single groups inhabited S1 and S3. However, S1 left its 1.6-ha fragment for about 35% of the study days to visit a 2-ha portion of a *ca*. 10-ha fragment used by other five groups. We inferred the presence of *ca*. 12, 13, and 18 social groups in L1, L2, and L3, respectively, based on estimates of howler density (Morro São Pedro: 0.99 inds/ha [51], State Park of Itapuã: 0.75 inds/ha [52]) and the average group size at the study sites [*i*.*e*., 6 inds/group: 51, 52]. We identified study groups based on their composition and body characteristics (*e*.*g*., body size, hair color, and face scars) of certain group members.

### Behavioral Records

We studied the behavior of brown howlers during a 36-mo period (June 2011 to June 2014) in all fragments, except L2. The L2 group was studied during a 33-mo period (October 2011 to June 2014). Data were collected from dawn to dusk during four to five consecutive days for each group on a bimonthly basis using the instantaneous scan sampling method [53] with the aid of high-resolution binoculars (Swarovski® SLC 10 x 42). We used scan samples of 5 min at 15-min intervals. As required by this method, we sought each group member throughout the 5 min of the sampling unit. After that time, the sampling unit was finished regardless of the number of recorded group members. Then, a new scan sampling unit was conducted 10 min after the end of the previous one. Observations were concentrated on conspicuous adult, subadult, and juvenile individuals of both sexes because dependent and independent infants were difficult to observe and rarely fed independently. We recorded the following information during feeding: the plant items eaten (*i*.*e*., ripe and unripe fruit, mature and young leaves, leaf buds, flowers, and flower buds), the plant species, and the growth form (*i*.*e*., tree, palm, vine, and liana).

Overall, we collected 35,514 behavior records from groups in small fragments and 30,688 records in large fragments (S3 Table). Feeding accounted for 20% to 26% of group records in small fragments and for 17% to 20% in large fragments (S3 Table). The contribution of each plant species, item, and growth form to the diet was estimated as the percentage of feeding records devoted to it. We calculated the number of species composing the diet of each group based on rarified data (1,700 scan records per group) to deal with differences in sampling effort among groups (S3 Table). The same procedure was used to calculate the intermonth diet similarity described below. Finally, we believe that differences in handling time among food items might have a lower influence on the pattern found in our study because brown howlers also exploited large fruits that require considerable handling (*e*.*g*., *Syagrus romanzoffiana*, *Inga* spp., and the alien species *Diospyros kaki*). Additionally, the potential effect of handling time on results must be minimized because individuals feed alternatively on different food items. Therefore, the likelihood of recording any item might be proportional to its actual contribution to the diet.

### Food Availability

To determine whether diet composition responded to resource availability, we first estimated the local availability (*i*.*e*., density, distribution, and abundance) of tree resources. For this, we performed tree surveys in the home range of study groups. We established fifteen 100 x 5 m linear transects (= 7,500 m^2^) in each site, where we identified all trees ≥5 cm diameter at breast height (DBH). The identification was based on taxonomic keys of the flora of Rio Grande do Sul State [50]. Voucher samples of trees that were not identified in the field were collected for identification with the help of an expert botanist (C.A. Mondin).

We calculated the importance value index (IVI) of each species as a measure of availability [54]. This index is calculated by the sum of density (number of trees of a given species/7,500 m^2^), frequency (number of transects in which the species was found/15 transects), and dominance (total basal area of the species in the 7,500 m^2^).

We estimated the temporal changes in resource availability of each site by randomly assigning 4 to 10 adult trees of the 20 highest-ranking native top food species for howler monkeys in southern Brazil (reviewed by [40]) for phenological monitoring. We estimated the amount of fruit (ripe and unripe), flowers (open and buds), and leaves (mature, young, and buds) one day before the beginning of each group’s bimonthly follow. We assigned a value ranging from 0 to 4 depending on the intensity or percentage of tree crown covered by a particular phenophase according to the Fournier semi-quantitative method [55]. We also conducted the phenological sampling with the help of high-resolution binoculars. Following Agostini et al. [56], we averaged the phenological scores of individual trees of each top food species to obtain a Phenological Index for the Species (PIS) for each month and phenophase. Therefore, we obtained a monthly Food Availability Index (FAI) for each top food species by multiplying its PIS by its IVI [56]. Finally, we calculated a monthly total FAI for each phenophase in each study site by summing up the FAI indices of all top food species.

### Statistical Analyses

We pooled the data from all sampling months to estimate the overall plant species richness of the diet of each study group. We estimated the expected number of plant species in the diet using four nonparametric estimators (ICE, ACE, Chao2, and Jack1) with EstimateS v.9.1.0 [57] to assess the completeness of the list of food resources exploited by groups. We used the mean of these estimators to calculate the proportion of species recorded in the samples (*i*.*e*., observed richness/mean of estimators). We assumed that our sampling effort was sufficient to record the bulk of the diet because the mean ± SD sample coverage per site was 77% ± 7% (S4 Table). We computed individually-based rarefaction curves to compare diet richness among study groups using 95% confidence intervals of the moment-based estimator (Sobs Mao Tau) [58]. Non-overlapping confidence intervals indicated statistically significant differences in richness [58].

To assess whether intermonth diet similarity differed between groups, we first calculated intermonth Morisita-Horn similarity indices for each group using EstimateS v.9.1.0 [57]. Then, we used rarified data for running generalized linear models (GLM) with quasibinomial error distribution and logit link-function as suggested for proportion data [59]. Differences between study groups were identified using post-hoc contrasts over the function ‘glht’ of the R package multcomp [60]. We used Chi-square tests of goodness-of-fit to compare the rarified number of feeding records devoted to each plant item (*i*.*e*., mature leaves, young leaves, ripe fruits, and unripe fruits) or growth form by each group. We used Bonferroni correction over the function ‘p.adjust’ because of multiple comparisons of the same data sets. Finally, we used linear regressions of log-transformed data to assess whether the availability of ripe fruit, young leaves, and flowers in each fragment was a good predictor of their consumption by study groups. Data were tested for normality and homocedasticity using the Shapiro-Wilk and the Levene tests provided by the package outliers prior to running these analyses. All statistical analyses were run in R v.3.2.1 [60]. Two-tailed *P-*values are reported for all tests because significant results in the opposite direction of our expectations are also ecologically relevant.

## Results

### Diet Richness and Top Food Species

**Fig 2:**
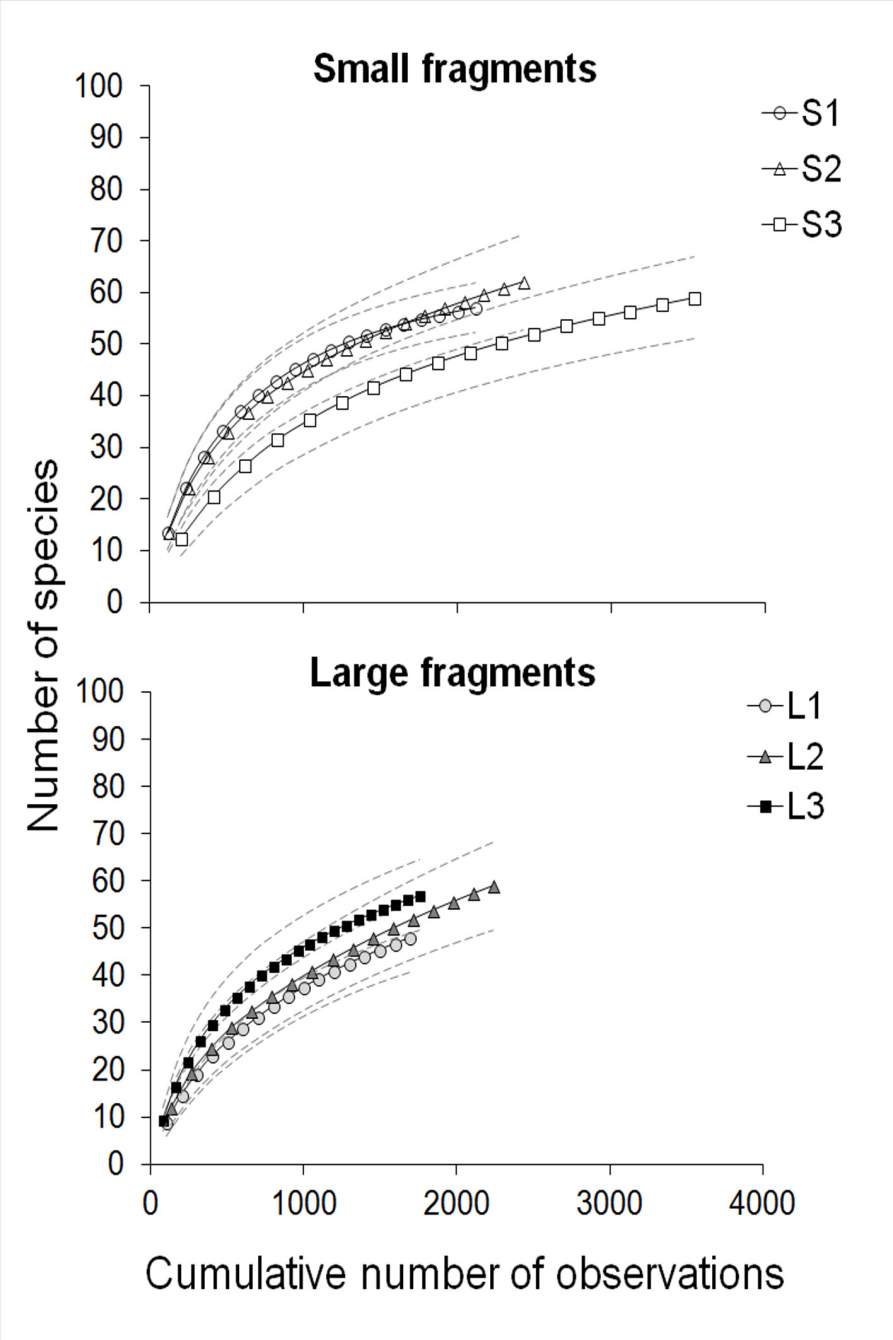
Individual-based rarefaction curves of the number of plant species used as food sources by brown howler monkeys in six study sites in the State of Rio Grande do Sul, Brazil. Curves for the small fragments (A) and the large fragments (B) are shown. Dashed lines indicate 95% confidence intervals.

Based on the rarified data, the diet of brown howlers inhabiting small fragments was composed of 45, 54, and 55 plant species (mean ± SD = 51 ± 5, total = 91 species) belonging to 72 genera and 43 families. Groups inhabiting large fragments exploited 48, 51, and 56 plant species (mean ± SD = 52 ± 4, total = 87 species) belonging to 67 genera and 39 families (S4 Table). However, the non-parametric estimators suggest that expected richness ranged from 64 (S1) to 88 species (L2, S4 Table; the recorded diet richness of each group increases when all data are taken into account, see S5 Table). Diet richness was similar in small (Fig 2A) and large (Fig 2B) fragments. These findings support our first prediction.

The number of top food species was 9, 16, and 16 (mean ± SD = 14 ± 4, total = 25 species) in small fragments and 10, 11, and 15 (mean ± SD = 12 ± 3, total = 22 species) in large fragments (Table 1). Groups inhabiting fragments of similar size shared about a quarter of top food species (small: 6/25 = 24%; large: 6/22 = 27%; Table 1). All groups shared four top food species (*Ficus cestrifolia*, *Coussapoa microcarpa*, *Cuspidaria convoluta*, and *Guapira opposita*, Table 1). Most top food species were large trees that bear fleshy fruit, such as *F*. *cestrifolia*, *F*. *luschnathiana*, *C*. *microcarpa*, *G*. *opposita*, and *Diospyros inconstans*. However, some of them produce dry fruit, such as *Machaerium stipitatum* and *Luehea divaricata* (Table 1). A high proportion of feeding records in both small (18%, 20%, and 45%) and large (20%, 24%, and 25%) fragments was devoted to plants of the family Moraceae, particularly because of the contribution of *F*. *cestrifolia* (Table 1).

**Table 1:** Percentage of total feeding records and importance value index (in parentheses) for the top food species in the diet of brown howlers in each study fragment. Growth form (GF) also shown.

			Study group					
* *Family	Species^a^	GF	S1	S2	S3	L1	L2	L3
Moraceae	*Ficus cestrifolia* (FL,UF,RF,YL)	Tree	12.3 (8.5)	15.5 (8.4)	41.0 (9.1)	21.2 (3.2)*	20.4 (5.6)*	20.7 (4.4)*
Urticaceae	*Coussapoa microcarpa* (FL,UF,RF,YL,ML)	Tree	1.7 (1.0)	10.8 (6.8)	11.0 (4.8)	13.1 (7.7)*	5.8 (5.4)	8.2 (7.3)
Bignoniaceae	*Cuspidaria convoluta* (FL,YL,ML)	Vine	5.3	10.6	11.3	9.6	14.5	5.0
Nyctaginaceae	*Guapira opposita* (FL,UF,RF,YL,ML)	Tree	4.2 (23.7)	4.4 (6.9)	2.9 (11.4)	7.1 (20.2)	11.7 (24.3)	6.4 (47.5)
Rutaceae	*Zanthoxylum rhoifolium* (RF,YL,ML)	Tree	17.3 (1.4)*	5.1 (3.2)	2.8 (2.1)	__	__	2.4 (1.7)
Ebenaceae	*Diospyros inconstans* (FL,UF,RF,YL,ML)	Tree	4.3 (6.5)	__	__	11.5 (8.4)	2.0 (11.8)	5.2 (11.0)
Arecaceae	*Syagrus romanzoffiana* (FL,UF,RF)	Palm	4.2 (3.1)	__	__	6.3 (3.5)*	8.1 (1.9)*	5.1 (1.2)
Moraceae	*Sorocea bonplandii* (UF,RF,YL)	Tree	2.8 (2.4)	2.3 (1.2)	1.6 (4.2)	3.4 (9.8)	__	__
Anacardiaceae	*Lithraea brasiliensis* (UF,RF,YL,ML)	Tree	1.8 (28.8)	__	3.6 (10.3)	__	3.8 (18.0)	2.0 (2.8)
Fabaceae	*Machaerium stipitatum* (YL,ML)	Tree	8.4 (6.0)	2.6 (0.8)	__	__	__	10.4 (7.1)
Moraceae	*Ficus luschnathiana* (FL,UF,RF,YL,ML)	Tree	4.7 (1.7)	__	2.8 (0.1)*	__	__	3.4 (0.1)*
Malvaceae	*Luehea divaricata* (FL,UF,YL,ML)	Tree	__	3.1 (9.5)	__	2.8 (5.0)	__	1.7 (12.8)
Myrtaceae	*Psidium guajava* (UF,RF)^b^	Tree	5.1 (0.1)*	1.7 (0.1)*	__	__	__	__
Fabaceae	*Enterolobium contortisiliquum* (FL,UF,RF,YL,ML)	Tree	2.5 (9.3)	__	__	__	4.2 (4.9)	__
Sapotaceae	*Chrysophyllum gonocarpum* (FL,UF,RF,YL,ML)	Tree	__	10.1 (0.1)*	__	__	__	2.4 (0.6)
Myrtaceae	*Campomanesia xanthocarpa* (UF,RF)	Tree	3.4 (2.0)	__	__	__	__	__
Apocynaceae	*Forsteronia leptocarpa* (ML,YL)	Vine	1.6	__	__	__	__	__
Euphorbiaceae	*Mandevilla coccinea* (YL,ML)	Vine	1.5	__	__	__	__	__
Salicaceae	*Banara parviflora* (FL,RF,YL,ML)	Tree	__	4.6 (4.2)	__	__	__	__
Fabaceae	*Dalbergia frutescens* (YL,ML)	Vine	__	2.9	__	__	__	__
Rosaceae	*Eriobotrya japonica* (UF,RF)	Tree	__	2.8 (0.1)*	__	__	__	__
Ebenaceae	*Diospyros kaki* (RF)^b^	Tree	__	2.6 (0.1)*	__	__	__	__
Convulvulaceae	*Ipomoea alba* (FL,YL,ML)	Vine	__	2.1	__	__	__	__
Myrtaceae	*Myrcia glabra* (FL,UF,RF)	Tree	__	__	3.6 (14.1)	__	__	__
Menispermaceae	*Hyperbaena domingensis* (UF,RF,YL,ML)	Vine	__	__	__	__	__	3.4
Erythroxylaceae	*Erythroxylum argentinum* (FL,UF,RF,YL)	Tree	__	__	__	__	__	2.1 (5.1)
Cannabaceae	*Celtis iguanaea* (RF,YL,ML)	Liana	__	__	__	__	__	1.9
Annonaceae	*Annona sylvatica* (UF,RF,YL,ML)	Tree	__	__	__	3.8 (7.5)	__	__
Clusiaceae	*Garcinia gardneriana* (UF,RF)	Tree	__	__	__	2.9 (8.9)	__	__
Lauraceae	*Ocotea porosa* (UF,RF,YL,ML)	Tree	__	__	__	__	5.2 (20.7)	__
Asteraceae	*Mikania glomerata* (YL,ML)	Vine	__	__	__	__	2.4	__
Sapindaceae	*Allophylus edulis* (FL,UF,RF,YL)	Tree	__	__	__	__	2.0 (20.9)	__
	**Σ species**	**33 **	**16**	**16**	**9**	**10**	**11**	**15**

* Preferred food species: species exploited in a proportion significantly higher than their availability in the environment.

^a^ Plant items: ripe fruit (RF), unripe fruit (UF), mature leaves (ML), young leaves (YL), and flowers (FL).

^b^ Alien species.

### Alien Species in Diet

Whereas four to six alien species were exploited as food sources in small fragments (total = 9 spp.), contributing 2.7% (S3) to 7.3% (S2) of total feeding records (Table 2), none was exploited in large fragments. The three most consumed alien species were guava (*Psidium guajava*), the Japanese persimmon (*Diospyros kaki*), and the loquat (*Eriobotrya japonica*). The first two were also top food species for groups S1 and S2 (Table 1). These results lend support to our second prediction.

**Table 2:** Percentage of total feeding records on the alien tree species (*N* = 9) exploited by brown howler monkeys in small fragments.

		Study group		
Family	Species/plant item^a^	S1	S2	S3
Myrtaceae	*Psidium guajava* (RF)	5.0	0.1	0.8
Rosaceae	*Eriobotrya japonica* (UF, RF)	0.3	2.8	0.3
Ebenaceae	*Diospyros kaki* (RF)	__	2.5	0.2
Rhamnaceae	*Hovenia dulcis* (RF)	0.5	1.6	__
Rutaceae	*Citrus reticulata* (RF)	1.0	0.2	__
Myrtaceae	*Syzygium cummini* (RF)	__	__	1.1
Moraceae	*Morus nigra* (YL, ML)	__	__	0.2
Araucariaceae	Araucaria angustifolia* (Seeds)	__	<0.1	0.1
Meliaceae	*Melia azedarach* (RF)	__	<0.1	__
	**Σ species**	**4**	**6**	**6**
	**% of total feeding records**	**6.8**	**7.3**	**2.7**

^a^ Plant items: ripe fruit (RF), unripe fruit (UF), mature leaves (ML), young leaves (YL).

* Conifer native to Brazil, but alien, cultivated in Porto Alegre, Rio Grande do Sul State.

### Diet Similarity

**Fig 3:**
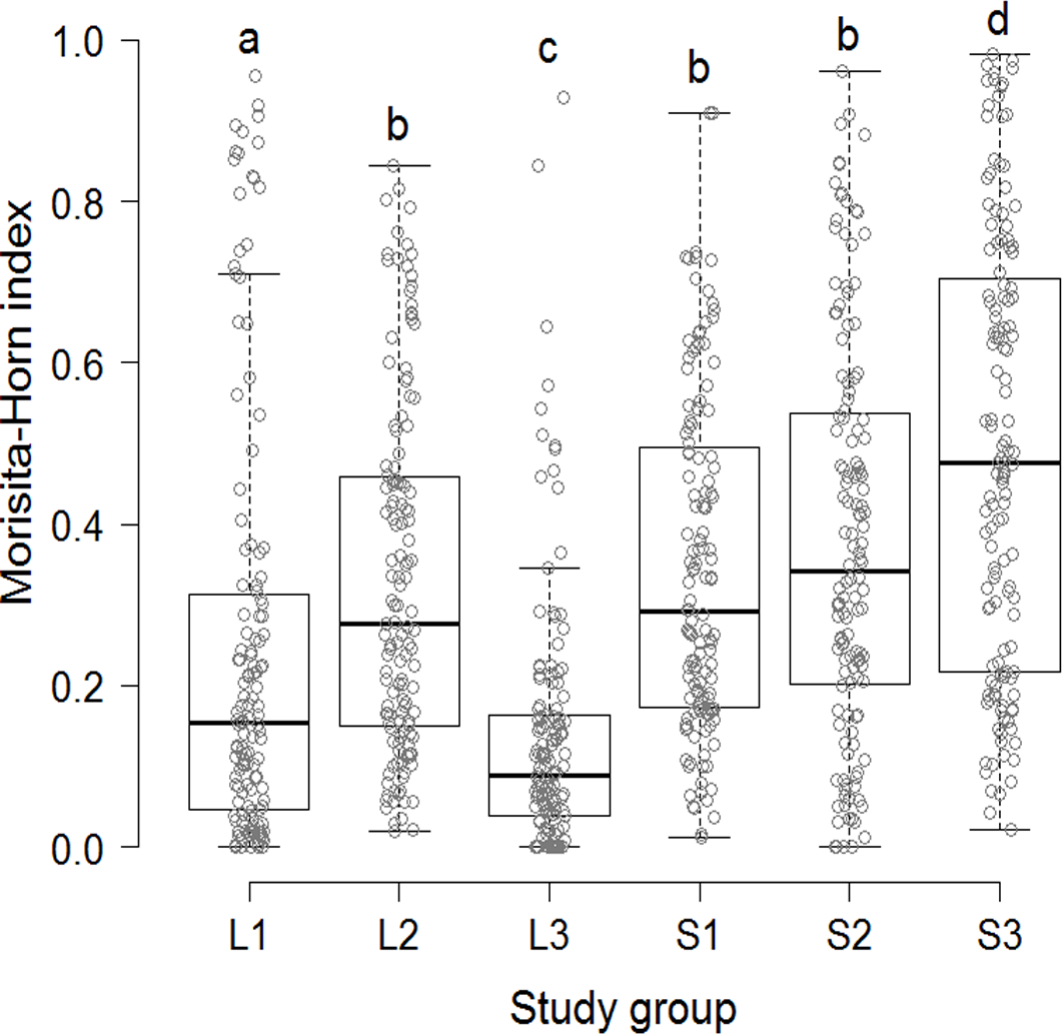
Intermonth diet similarity between study groups inhabiting small and large fragments. The line within a box represents the median of the Morisita-Horn index, the box represents the 25% and 75% interquartiles (IQR), and the whiskers represent the IQR multiplied by 1.5. Dots represent the actual data points for each group. Different letters indicate significant differences (*P*<0.05).

Intermonth diet similarity differed among study groups (GLM, *F*_5,810_ = 36.2, *P*<0.0001). It was higher in L2 than in L1 and L3, and higher in S1 and S2 than in S3 (contrast test, *P*<0.05 in all cases, Fig 3). In most cases, diet similarity was higher in small than in large fragments. Group L2 was an exception, as its diet similarity did not differ from those of groups S1 and S2 (Fig 3). These findings support the third prediction.

### Diet Composition

Overall, the same number of plant species (44) provided ripe fruit for howlers in small (27, 29, and 21 spp. in each group, respectively) and large (29, 20, and 30 spp., respectively) fragments, whereas 61 (43, 40, and 42 spp.) were exploited for young leaves in small fragments and 46 (35, 27, and 33 spp.) in large ones. Ripe fruit (25–41% of total feeding records) was the predominant plant item in the diet of all groups, except S1, followed by mature leaves (22–33%), young leaves (6–21%), unripe fruit (6–20%), flowers (1–10%), leaf buds (1–8%), and flower buds (2–5%).

**Fig 4:**
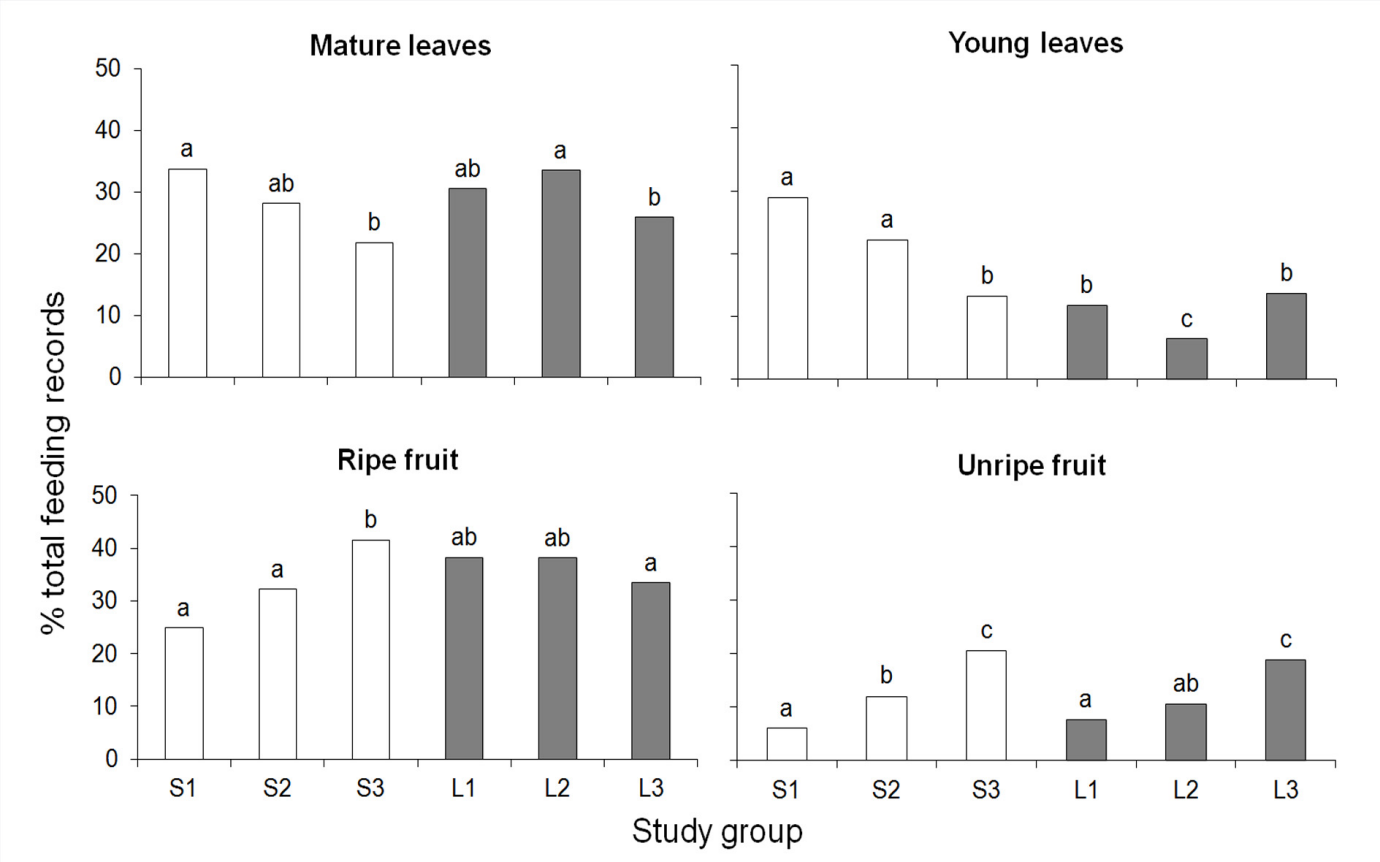
Percentage of feeding records devoted to each plant item in the diet of brown howler monkeys in large and small Atlantic forest fragments. Different letters above bars indicate significant differences (*P*<0.05).

The percentage of feeding records (hereafter referred to as consumption) devoted to all major food items differed among groups (mature leaves: *χ*^*2*^ = 41.2, young leaves: *χ*^*2*^ = 295.1, ripe fruit: *χ*^*2*^ = 73.7, unripe fruit: *χ*^*2*^ = 200.7, all df = 5, *P*<0.0001; contrast tests, *P*<0.05 in all significant cases; Fig 4). The consumption of mature leaves was lower in group S3 than in S1 and L2, whereas the consumption of young leaves was, in general, higher in groups inhabiting small than large fragments. While group S3 consumed more ripe and unripe fruit than the other groups inhabiting small fragments (probably due to the high consumption of figs, see Table 1), the consumption of these items by the groups living in large fragments was less variable (Fig 4). Therefore, our fourth prediction is only partially supported for young leaves.

**Fig 5:**
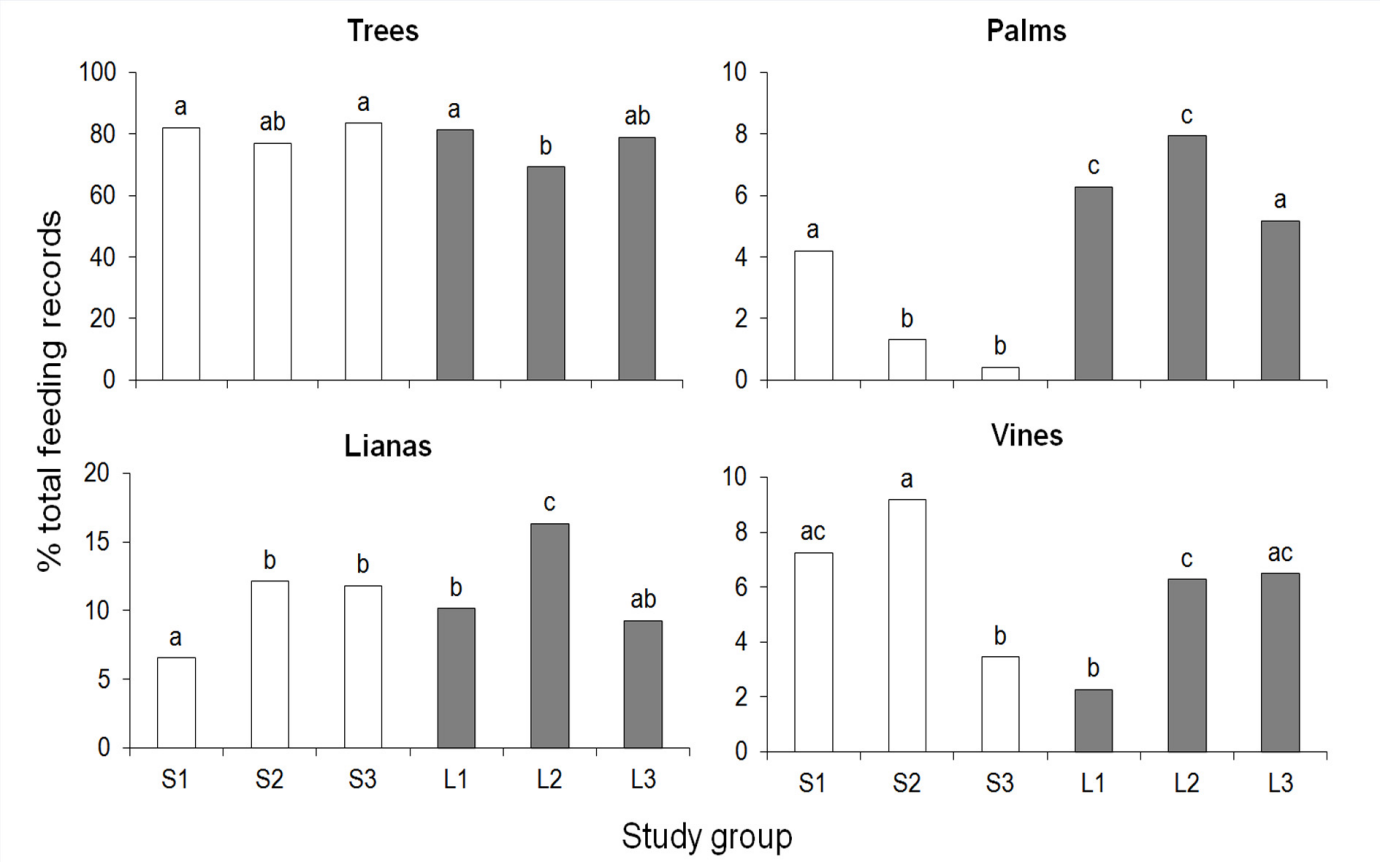
Percentage of feeding records devoted to each growth form in the diet of brown howler monkeys in large and small Atlantic forest fragments. Different letters above bars indicate significant differences (*P*<0.05).

A total of 63 tree species, 5 lianas, 1 palm, and 22 vines provided food in small fragments, whereas these figures were 56, 6, 1, and 24 species in large fragments. Trees were the most exploited growth form in all fragments (69–82% of total feeding records), followed by lianas (6–16%), vines (2–8%), and palms (<1–8%). The contribution of these growth forms as food sources varied noticeably among groups (trees: *χ*^*2*^ = 24.2, *P*<0.001, palm: *χ*^*2*^ = 144.0, *P*<0.0001, lianas: *χ*^*2*^ = 47.1, *P*<0.0001, vines: *χ*^*2*^ = 80.0, *P*<0.0001, all df = 5; contrast tests, *P<*0.05 in all significant cases, Fig 5). Whereas the contribution of trees was lower in L2 compared with S1, S3 and L1, the consumption of fruits and flowers of the unique palm exploited, *S*. *romanzoffiana*, was often higher in large than in small fragments despite the similarity of its IVI and relative density at the study sites (S6 Table). Although there were group differences in the exploitation of lianas and vines, there was no clear trend related to fragment size (Fig 5). Therefore, our fifth prediction is not supported.

### Food Availability and Item Consumption

The availability of ripe fruit and young leaves from native top food species was a good predictor of their consumption by some study groups. In the case of flowers, the relationship only approached significance for groups S2 and L3 (Table 3). Most significant relationships (4 of 6) were found in large fragments (Table 3; see also S1–S3 Figs). The influence of availability on consumption resulted in a high intermonth variability in the contribution of each seasonal plant item to the diet of study groups (ripe fruit: 0–91%, young leaves: 0–62%, flowers: 0–41%). Therefore, our last prediction is also partially supported.

**Table 3:** Linear regressions between the availability (independent variable) and the consumption (dependent variable) of seasonal plant items of the top food species by the brown howler monkey study groups.

Plant item	Small fragments	N	*R*^*2*^	*P*	Large fragments	N^a^	*R*^*2*^	*P*
Ripe fruits	S1	14	0.04	0.51	L1	15	0.51	**<0.01**
	S2	14	0.17	0.12	L2	15	0.43	**<0.01**
	S3	15	0.48	**<0.01**	L3	14	0.02	0.61
Young leaves	S1	14	0.46	**<0.01**	L1	15	0.4	**0.01**
	S2	14	0.1	0.26	L2	15	0.15	0.16
	S3	15	0.17	0.16	L3	14	0.45	**<0.01**
Flowers	S1	14	0.12	0.22	L1	15	0.01	0.67
	S2	14	0.28	0.05	L2	15	0.04	0.45
	S3	15	0.03	0.55	L3	14	0.24	0.07

^a^ N = number of study months. Significant correlations highlighted in bold.

## Discussion

The dietary flexibility of tropical primates facing recent increases in habitat deterioration [9, 61] has grown as a research topic in the last decades [4, 21, 22, 30]. However, our knowledge on the feeding strategies adopted by primates in response to seasonal and habitat-related reductions in food availability is incipient at best [21, 62], even for well-studied genera, such as *Alouatta* [20, 35]. In this respect, we found that brown howlers alter their diet in response to local and seasonal changes in food availability by adopting flexible strategies. These strategies included the consumption of plant items from alien species and a higher consumption of young leaves in small fragments. On the other hand, groups inhabiting large fragments showed a higher consumption of palm fruit and were capable of exploiting seasonal resources (especially ripe fruit and young leaves) according to their availability.

Overall diet richness was similar in small and large fragments as expected. Studies indicate that primates inhabiting small and/or low quality forests can compensate the lower density and/or richness of native tree species used as food sources by exploiting alien plant species that are common in the surrounding anthropogenic matrix [32, 33, 43, 44, 63]. We found that alien species, especially those producing edible fruit, such as *Psidium guajava*, *Diospyros kaki*, and *Eriobotrya japonica*, were important supplements to the diet of brown howlers inhabiting small fragments. However, the ability to exploit alien species might incur high costs. Howlers face high risks of electrocution and predation by domestic animals and humans near human settlements (ÓMC, pers. obs.), where these resources are more common. In fact, most alien trees exploited by our brown howler groups were in gardens and orchards, places inhabited by one to five dogs. Long-term monitoring is necessary for evaluating whether this behavioral plasticity in anthropogenic habitat patches increases or decreases the fitness of brown howlers.

Although brown howlers inhabiting small fragments supplemented their diet with alien species and exploited a richer diversity of leaf sources, these strategies were not sufficient to overcome some consequences of living in a habitat with a less diverse plant assemblage (see S1 Table). Compared with groups inhabiting large fragments, they presented a higher intermonth diet similarity. Whether this higher similarity imposes significant health costs to individuals is unknown.

Increasing leaf consumption is another frequent response of primates to food scarcity in small and/or disturbed habitats [27, 29, 30, 40, 63]. We found support for this trend, at least for the consumption of young leaves. In addition to this increase in the contribution of young leaves to the diet, groups inhabiting small fragments also tended to exploit a more diverse array of sources of this item. Differences in food availability cannot account for this result because large fragments showed higher tree species richness than small ones. The contribution of lianas and vines also cannot explain it because the diversity of these non-tree growth forms in the diet of groups was quite similar in both habitats (contrary to the pattern found for other atelids [26, 27, 45]). Therefore, it is possible that the greater exploitation of sources of young leaves in small fragments is a strategy for optimizing nutrient acquisition and/or avoiding the overconsumption of the same secondary metabolites [62, 64]. The finding that intermonth diet similarity is higher in these fragments is apparently incompatible with the latter unless the most commonly exploited species contain less secondary metabolites than those avoided. Therefore, studies comparing the nutritional and toxic contents of eaten and avoided young leaves and fruits are necessary to improve our understanding of the factors that drive howler monkey food selection in these habitats.

Contrary to our expectation, the percentage of feeding records devoted to the consumption of fruit was similar between small and large fragments. The aforementioned exploitation of alien fruit explains this finding together with the high consumption of figs (*Ficus* spp.), particularly in S3. The importance of *Ficus* spp. as keystone species for howler monkeys and other atelids has been largely recognized [20, 23, 40, 65] and related to their high nutritional value [66], asynchronous fruiting [67, 68], and the genus’s wide geographic distribution [67]. In this respect, we found that *F*. *cestrifolia* bears immature and/or mature fruit throughout the year, whereas fruiting of the other top food species is restricted to a period of 2.5 to 5 months each year. Therefore, the almost permanent availability of figs potentially minimizes nutritional stress in small fragments.

Whereas the exploitation of trees, lianas, and vines by our study groups did not show a pattern related to fragment size, groups inhabiting large fragments showed a higher ingestion of *S*. *romanzoffiana* fruit. The IVI and relative density of this palm, an important top food species for brown howlers [40], was similar in both habitats. This finding contradicts the expectation that palms (as well as lianas and vines) are more abundant in small fragments and/or disturbed habitats [26, 31, 45]. In fact, the absolute number of adult palms is remarkably higher in large fragments because of differences in area (S6 Table) and because the processes of seed dispersal and seedling recruitment of this large-seeded (2–3 cm in length) species is severely compromised in small and/or defaunated habitats [69]. This higher availability certainly explains the species’ greater importance to the diet of howlers in large fragments. The lack of data on the density and richness of climbers in the study sites, on the other hand, hampers us from evaluating why their contributions to the diet of our groups did not differ.

We also confirmed that the availability of seasonal items from top food species influenced howler monkey consumption, especially in large fragments. As a consequence, the level of folivory and frugivory may vary sharply among months, years, and habitat types (e.g., [20, 48, 62]. For instance, we found that brown howlers are strongly frugivorous (>90% of total feeding records) during fruiting peaks, but can switch to a predominantly lower-energy, leafy diet (>60%) in periods of fruit scarcity. This concurs with the statement that howlers are as frugivorous as possible and as folivorous as necessary (sensu [70]). At least two non-mutually exclusive hypotheses might explain the weaker relationship between availability and consumption found for groups in small fragments. Brown howlers inhabiting these fragments may compensate the lower availability of seasonal items of top food species by increasing the percentage of feeding records devoted to non-preferred or fallback foods as shown for other primates [28, 31, 62, 63, 71]. Otherwise, as mentioned above, alien species may offer alternative high-quality foods. In fact, the availability of fruit from the six most important alien species (not included in our phenological monitoring) clearly influenced their consumption in small fragments (ÓMC, pers. obs.).

In sum, we showed that brown howlers varied their food choices in response to local and seasonal variations in resource availability, supporting the importance of dietary switching for primates inhabiting seasonal [21, 25, 62] and/or disturbed habitats [4, 18, 27, 29]. Although this flexibility allows individual howlers to thrive in quite small and/or disturbed fragments, the long-term survival of populations living in these environments is uncertain [40]. This is particularly critical if we take into account present and prospective human modifications of tropical forests [9, 10]. Therefore, studies comparing the health and the fecundity rate of brown howlers in small and large Atlantic forest fragments with contrasting levels of disturbance are necessary to assess the long-term demographic consequences of this dietary flexibility.

## Supporting Information

S1 Fig Seasonal availability and consumption of ripe fruit by brown howler monkeys. (DOCX)

S2 Fig Seasonal availability and consumption of young leaves by brown howler monkeys. (DOCX)

S3 Fig Seasonal availability and consumption of flowers by brown howler monkeys. (DOCX)

S1 Table Importance value index (and relative density of trees in inds/ha) of food tree species exploited by brown howler monkeys in each fragment. (DOCX)

S2 Table Age-sex composition of the study groups in December 2013. (DOCX)

S3 Table Sampling effort on each group during the study period (June 2011-June 2014) and percentage of behavioral records devoted to feeding. (DOCX)

S4 Table Observed and expected number of species in each study fragment in southern Brazil based on scan sampling records. (DOCX)

S5 Table Observed and expected number of species in each study site in southern Brazil based on records from both scan and focal-animal sampling. (DOCX)

S6 Table Relative and absolute density of adult individuals of the palm *Syagrus romanzoffiana* in each study site. (DOCX)
