# Supplementary material for: Feeding Strategies of Brown Howler Monkeys in Response to Variations in Food Availability
Source: PLoS One. 2016 Feb 5;11(2):e0145819. doi: 10.1371/journal.pone.0145819 (PMC4743924; doi:10.1371/journal.pone.0145819)
Supplement: S2 Fig — (DOCX) [file pone.0145819.s002.docx]

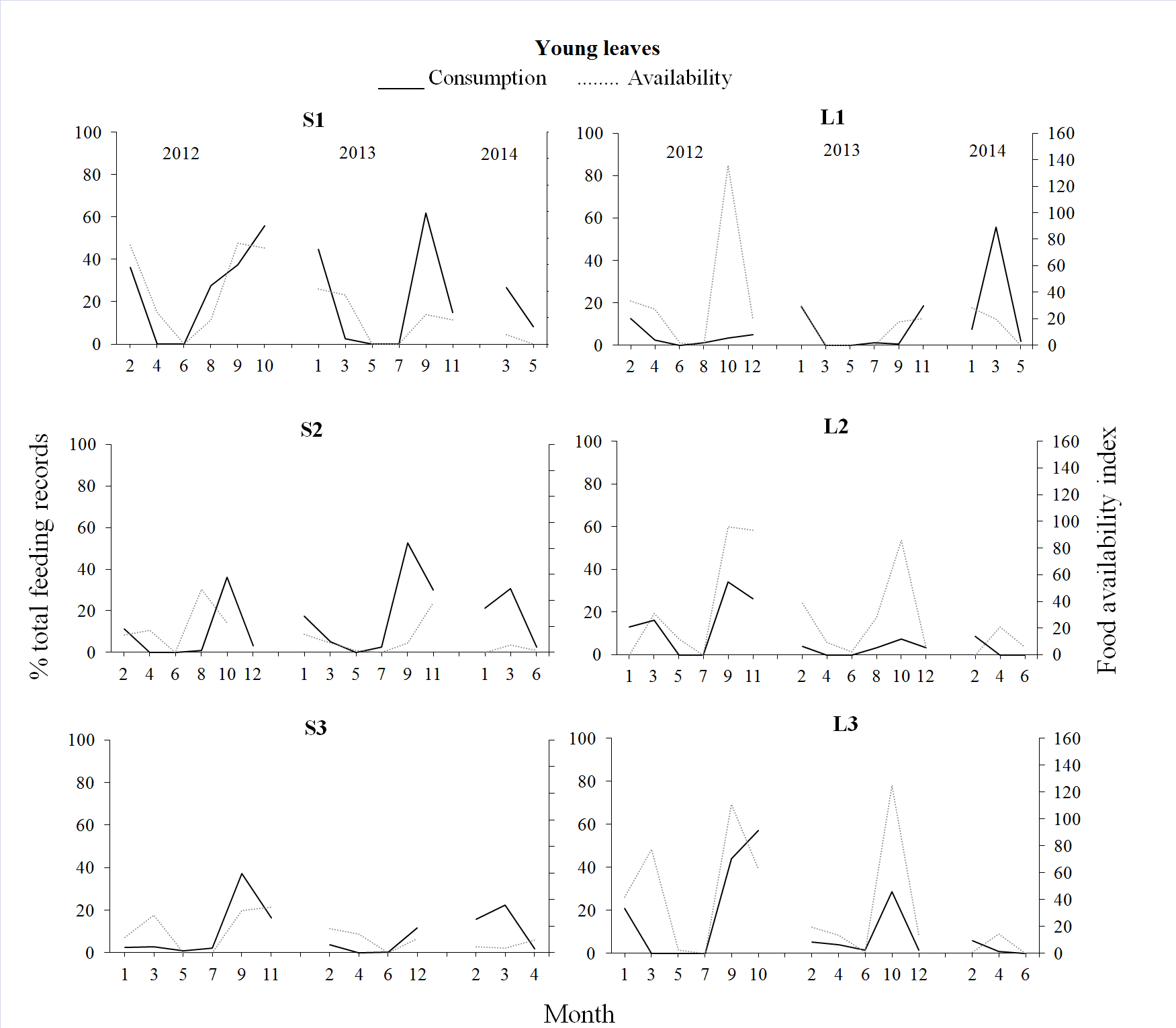


**S2 Fig. Seasonal availability and consumption of young leaves by brown howler monkeys.** Solid lines represent the percentage of total feeding records devoted to young leaves. Dashed lines represent food availability indices calculated as described in Methods. Results of linear regressions are shown in Table 3.
