# Supplementary material for: Feeding Strategies of Brown Howler Monkeys in Response to Variations in Food Availability
Source: PLoS One. 2016 Feb 5;11(2):e0145819. doi: 10.1371/journal.pone.0145819 (PMC4743924; doi:10.1371/journal.pone.0145819)
Supplement: S1 Table — (DOCX) [file pone.0145819.s004.docx]

**S1 Table. Importance value index (and relative density of trees in inds./ha) of food tree species exploited by brown howler monkeys in each fragment.** Data based on plant surveys of trees ≥5 cm diameter at breast height (DBH). Top food species marked with an asterisk.

| Family | Species | Study site | | | | | |
| --- | --- | --- | --- | --- | --- | --- | --- |
|  |  | S1 | S2 | S3 | L1 | L2 | L3 |
| Euphorbiaceae | *Sebastiania serrata* | 118.2 (37.5) | 122.5 (34.4) | 121.8 (33.5) | 31.6 (11.7) | 39.5 (14.5) | 50.2 (22.5) |
| Nyctaginaceae | *Guapira opposita* | 19.8 (7.9)* | 5.3 (1.6)* | 8.7 (2.9)* | 21.4 (6.3)* | 26.3 (9.3)* | 76.2 (17.2)* |
| Primulaceae | *Myrsine umbellata* | 23.4 (8.8) | 21.3 (7.2) | 27.6 (10.3) | 6.4 (2.0) | 18.6 (7.2) | 28.2 (10.1) |
| Salicaceae | *Casearia sylvestris* | 13.3 (5.2) | 32.8 (14.7) | 9.9 (3.6) | 5.3 (1.7) | 13.8 (5.9) | 16.7 (8.2) |
| Sapindaceae | *Allophylus edulis* | 10.3 (3.8) | 8.3 (3.6) | 10.6 (3.8) | 12.8 (3.6) | 21.1 (7.7)* | 7.4 (2.5) |
| Anacardiaceae | *Lithraea brasiliensis* | 26.3 (8.7)* | 11.4 (4.1) | 7.5 (2.4)* | 3.7 (1.2) | 17.4 (5.7)* | 2.0 (0.7)* |
| Sapotaceae | *Chrysophyllum marginatum* | 12.3 (4.2) | 3.0 (0.9) | 6.3 (2.1) | 12.8 (3.2) | 10.7 (3.7) | 12.7 (5.2) |
| Rubiaceae | *Faramea montevidensis* | 0.6 (0.1) | 14.8 (8.4) | 26.7 (15.5) | 0.5 (0.1) | 1.0 (0.2) | 2.9 (1.3) |
| Ebenaceae | *Diospyros inconstans* | 5.2 (1.8)* | 2.4 (0.6) | 1.0 (0.3) | 6.8 (2.1)* | 10.0 (3.7)* | 8.7 (3.1)* |
| Moraceae | *Sorocea bonplandii* | 2.2 (0.5)* | 0.9 (0.2)* | 3.7 (1.0)* | 8.7 (3.5)* | 6.6 (2.7) | 7.8 (3.9) |
| Moraceae | *Ficus cestrifolia* | 6.0 (1.0)* | 5.2 (1.0)* | 5.7 (1.2)* | 2.2 (0.6)* | 4.1 (0.8)* | 2.5 (0.4)* |
| Urticaceae | *Coussapoa microcarpa* | 0.7 (0.1)* | 4.8 (1.4)* | 3.0 (0.8)* | 6.1 (1.6)* | 4.1 (1.4)* | 5.0 (1.6)* |
| Erythroxylaceae | *Erythroxylum argentinum* | 4.2 (0.9) | 1.9 (0.5) | 1.8 (0.7) | 7.0 (2.0) | 6.9 (2) | 3.7 (1.1)* |
| Salicaceae | *Casearia decandra* | 1.5 (0.4) | 5.5 (1.6) | 3.0 (0.8) | 2.5 (0.7) | 7.6 (2.8) | 2.2 (0.6) |
| Fabaceae | *Enterolobium contortisiliquum* | 7.0 (1.6)* | 3.5 (1.4) | 0.5 (0.1) | 0.6 (0.1) | 3.5 (1.1)* | 3.3 (0.8) |
| Myrtaceae | *Campomanesia xanthocarpa* | 1.3 (0.2)* | 0.5 (0.1) | 3.3 (1.2) | 1.2 (0.3) | 2.9 (1.6) | 4.1 (1.2) |
| Rutaceae | *Zanthoxylum rhoifolium* | 1.3 (0.2)* | 2.8 (0.8)* | 1.8 (0.7)* | 1.1 (0.2) | 3.3 (0.8) | 1.5 (0.3)* |
| Arecaceae | *Syagrus romanzoffiana*^1^ | 2.3 (0.6)* | 0.9 (0.3) | 1.0 (0.3) | 2.6 (0.7)* | 1.5 (0.3)* | 1.0 (0.2)* |
| Lauraceae | *Nectandra megapotamica* | 0.6 (0.1) | 1.6 (0.5) | 1.0 (0.3) | 1.2 (0.3) | 1.5 (0.3) | 2.0 (0.4) |
| Malvaceae | *Luehea divaricata* | 3.0 (0.6) | 6.8 (2.3)* | 10.1 (3.4) | 3.4 (0.9)* | ─ | 10.0 (3.2)* |
| Araliaceae | *Dendropanax cuneatus* | 1.4 (0.3) | 3.4 (1.0) | ─ | 0.6 (0.1) | 2.0 (0.4) | 1.0 (0.2) |
| Meliaceae | *Trichilia claussenii* | 1.3 (0.2) | 0.5 (0.1) | ─ | 57.5 (20.3) | 24.9 (11.3) | 7.3 (2.6) |
| Annonaceae | *Annona sylvatica* | 0.6 (0.1) | ─ | 1.0 (0.3) | 6.3 (2.1)* | 1.6 (0.4) | 2.7 (0.7) |
| Sapindaceae | *Cupania vernalis* | ─ | 0.7 (0.4) | 1.0 (0.3) | 29.6 (8.2) | 42.9 (13.1) | 4.5 (1.6) |
| Salicaceae | *Banara parviflora* | ─ | 3.0 (0.9)* | 10.3 (3.5) | 8.6 (2.6) | 1.1 (0.3) | 3.8 (1.3) |
| Fabaceae | *Mimosa bimucronata* | 6.3 (3.2) | 2.6 (1.1) | 1.0 (0.3) | ─ | ─ | 1.0 (0.2) |
| Fabaceae | *Inga striata* | 1.3 (0.2) | 0.5 (0.1) | 2.2 (0.7) | ─ | ─ | 5.9 (1.3) |
| Boraginaceae | *Cordia americana* | 0.7 (0.1) | 0.5 (0.1) | ─ | 6.8 (1.9) | 9.2 (2.2) | ─ |
| Lauraceae | *Ocotea pulchella* | 0.7 (0.1) | 0.9 (0.2) | ─ | 3.7 (0.9) | 5.3 (1.2) | ─ |
| Myrtaceae | *Myrcia glabra* | ─ | 10.4 (3.7) | 11.6 (4.2)* | ─ | 0.5 (0.1) | 4.3 (1.4) |
| Clusiaceae | *Garcinia gardneriana* | ─ | ─ | 1.1 (0.4) | 7.7 (3.3)* | 4.3 (1.0) | 3.4 (1.4) |
| Malvaceae | *Trema micrantha* | 1.3 (0.2) | 2.2 (0.8) | 0.5 (0.1) | ─ | ─ | ─ |
| Fabaceae | *Machaerium stipitatum* | 5.1 (1.3)* | 0.5 (0.1)* | ─ | ─ | ─ | 6.1 (2.0)* |
| Myrtaceae | *Psidium cattleianum* | 3.4 (1.2) | 0.5 (0.1) | ─ | ─ | ─ | 0.5 (0.1) |
| Moraceae | *Ficus luschnathiana* | 1.3 (0.2)* | ─ | ─* | 1.7 (0.4) | 2.1 (0.5) | ─* |
| Rosaceae | *Prunus myrtifolia* | ─ | 0.5 (0.1) | 1.5 (0.4) | ─ | 0.5 (0.1) | ─ |
| Boraginaceae | *Cordia ecalyculata* | ─ | 0.5 (0.1) | ─ | 0.5 (0.1) | 0.6 (0.2) | ─ |
| Sapotaceae | *Chrysophyllum gonocarpum* | ─ | ─* | 3.8 (1.4) | 0.5 (0.1) | ─ | 0.5 (0.1)* |
| Lauraceae | *Ocotea porosa* | ─ | ─ | 0.5 (0.1) | 6.1 (1.9) | 24.6 (5.2)* | ─ |
| Symplocaceae | *Symplocos uniflora* | 0.7 (0.1) | 0.9 (0.3) | ─ | ─ | ─ | ─ |
| Lauraceae | *Ocotea puberula* | 1.3 (0.2) | ─ | ─ | ─ | ─ | 2.7 (0.6) |
| Rutaceae | *Zanthoxylum fagara* | 0.7 (0.1) | ─ | ─ | ─ | 0.5 (0.1) | ─ |
| Anacardiaceae | *Schinus terebinthifolius* | ─ | 5.9 (2.0) | 1.5 (0.4) | ─ | ─ | ─ |
| Fabaceae | *Inga vera* | ─ | ─ | 2.9 (1.0) | ─ | ─ | 0.5 (0.1) |
| Cecropiaceae | *Cecropia peltata* | 1.6 (0.5) | ─ | ─ | ─ | ─ | ─ |
| Malvaceae | *Ceiba speciosa* | ─ | 0.9 (0.3) | ─ | ─ | ─ | ─ |
| Araucariaceae | *Araucaria angustifolia* | ─ | 0.6 (0.2) | ─ | ─ | ─ | ─ |
| Myrtaceae | *Myrcianthes pungens* | ─ | ─ | ─ | 14.5 (5.6) | ─ | ─ |
| Fabaceae | *Lonchocarpus nitidus* | ─ | ─ | ─ | 5.7 (3.5) | ─ | ─ |
| **Food sp. richness** |  | **37** | **40** | **37** | **35** | **34** | **37** |
| **TFS^1^ richness** |  | **12** | **8** | **8** | **9** | **9** | **12** |
| **Σ of IVI for TFS** |  | **78.5** | **29.3** | **42** | **65.2** | **112.6** | **121.9** |
| **Σ TFS densiy^4^** |  | **24.1** | **8.3** | **13.2** | **21.1** | **35.2** | **31.3** |

^1^ Top food species (TFS) are those that together contributed ≥80% of total feeding records to the diet of brown howlers during the study.

─ Species not recorded in the area sampled at the study site.
