# Supplementary material for: Feeding Strategies of Brown Howler Monkeys in Response to Variations in Food Availability
Source: PLoS One. 2016 Feb 5;11(2):e0145819. doi: 10.1371/journal.pone.0145819 (PMC4743924; doi:10.1371/journal.pone.0145819)
Supplement: S2 Table — (DOCX) [file pone.0145819.s005.docx]

**S2 Table. Age-sex composition of the study groups in December 2013**.

|  | Age-sex composition* | | | | | | | |
| --- | --- | --- | --- | --- | --- | --- | --- | --- |
| Fragment | AM | AF | SM | SF | JM | JF | I | Group size |
| S1 | 1 | 2 | 1 | - | 2 | - | - | **6** |
| S2 | 1 | 3 | 1 | - | 3 | 1 | 1 | **10** |
| S3 | 1 | 3 | 1 | - | 3 | - | 1 | **9** |
| L1 | 2 | 3 | 1 | - | 2 | - | 2 | **10** |
| L2 | 2 | 3 | - | - | 3 | - | 1 | **9** |
| L3 | 2 | 3 | - | 1 | 2 | 1 | - | **9** |
| Total | **9** | **17** | **4** | **1** | **15** | **2** | **5** | **53** |

*A=adult, S=subadult, J=juvenile, I=infant, M=male, F=female
