# Supplementary material for: Feeding Strategies of Brown Howler Monkeys in Response to Variations in Food Availability
Source: PLoS One. 2016 Feb 5;11(2):e0145819. doi: 10.1371/journal.pone.0145819 (PMC4743924; doi:10.1371/journal.pone.0145819)
Supplement: S3 Table — (DOCX) [file pone.0145819.s006.docx]

**S3 Table. Sampling effort on each group during the study period (June 2011-June 2014) and percentage of behavioral records devoted to feeding.**

| Sampling effort by group | | | | | |  | Feeding | | | |
| --- | --- | --- | --- | --- | --- | --- | --- | --- | --- | --- |
| Study site | Months | Days | Complete days | Hours | Scans | Records | Hours | Scans | Records | % records |
| Small | **60** | **193** | **132** | **1,468** | **5,873** | **35,514** | **560** | **2,238** | **8,097** | **23** |
| S1 | 21 | 67 | 45 | 492 | 1,968 | 9,868 | 180 | 719 | 2,117 | 21 |
| S2 | 19 | 61 | 40 | 438 | 1,750 | 11,949 | 156 | 624 | 2,436 | 20 |
| S3 | 20 | 65 | 47 | 539 | 2,155 | 13,697 | 224 | 895 | 3,544 | 26 |
| Large | **56** | **241** | **128** | **1,531** | **6,125** | **30,688** | **425** | **1,700** | **5,733** | **19** |
| L1 | 18 | 81 | 37 | 460 | 1,841 | 9,963 | 116 | 463 | 1,692 | 17 |
| L2 | 17 | 87 | 50 | 536 | 2,142 | 11,471 | 174 | 696 | 2,277 | 20 |
| L3 | 21 | 73 | 41 | 536 | 2,142 | 9,254 | 135 | 541 | 1,764 | 19 |
