# Supplementary material for: Feeding Strategies of Brown Howler Monkeys in Response to Variations in Food Availability
Source: PLoS One. 2016 Feb 5;11(2):e0145819. doi: 10.1371/journal.pone.0145819 (PMC4743924; doi:10.1371/journal.pone.0145819)
Supplement: S4 Table — (DOCX) [file pone.0145819.s007.docx]

**S4 Table. Observed and expected number of species in each study site in southern Brazil based on scan sampling records.**

| Group | Observed richness^a^ | Nonparametric richness estimator^b^ | | | |  |  |  |
| --- | --- | --- | --- | --- | --- | --- | --- | --- |
|  |  | ACE | ICE | Chao 2 | Jack1 | Mean | SD | % |
| S1 | 57 (54) | 60.7 | 65.7 | 62.7 | 68.3 | 64.3 | 3.3 | 88.6 |
| S2 | 62 (55) | 75.6 | 85.9 | 92.0 | 83.8 | 84.3 | 6.7 | 73.5 |
| S3 | 59 (45) | 61.8 | 77.2 | 78.8 | 78.8 | 74.1 | 8.2 | 79.6 |
| L1 | 48 (48) | 55.3 | 79.3 | 66.0 | 67.8 | 67.1 | 9.8 | 71.5 |
| L2 | 59 (51) | 70.8 | 103.6 | 91.3 | 85.3 | 87.8 | 13.6 | 67.2 |
| L3 | 57 (56) | 61.2 | 77.9 | 73.5 | 76.1 | 72.2 | 7.5 | 79.0 |

^a^ Rarified number of species based on 1,700 feeding records per group shown in parentheses.

^b^ The four non-parametric estimators are based on species incidence (presence/absence): average-based coverage estimator (ACE), incidence-based coverage estimator (ICE), Chao2, and Jackknife 1. The mean (±SD) of the four estimators, and the percentage of species recorded (observed species/mean of estimator x 100) are also shown as a measure of sampling completeness (%).
