# Supplementary material for: Feeding Strategies of Brown Howler Monkeys in Response to Variations in Food Availability
Source: PLoS One. 2016 Feb 5;11(2):e0145819. doi: 10.1371/journal.pone.0145819 (PMC4743924; doi:10.1371/journal.pone.0145819)
Supplement: S5 Table — (DOCX) [file pone.0145819.s008.docx]

**S5 Table. Observed and expected number of species in each study site in southern Brazil based on records from both scan and focal-animal sampling.**

| Group | Observed richness | Nonparametric richness estimator | | | |  |  |  |
| --- | --- | --- | --- | --- | --- | --- | --- | --- |
|  |  | ACE | ICE | Chao 2 | Jack1 | Mean | SD | % |
| S1 | 67 (63) | 77.4 | 78.1 | 79.9 | 83.1 | 79.6 | 2.5 | 84.1 |
| S2 | 77 (70) | 104.3 | 116.8 | 129.2 | 107.3 | 114.4 | 11.2 | 67.3 |
| S3 | 75 (56) | 93.4 | 112.1 | 133.6 | 105.2 | 111.0 | 16.9 | 67.5 |
| L1 | 57 (57) | 64.0 | 94.7 | 98.0 | 97.4 | 88.5 | 16.4 | 64.4 |
| L2 | 72 (65) | 82.4 | 106.0 | 94.0 | 78.3 | 90.2 | 12.5 | 79.9 |
| L3 | 61 (61) | 65.0 | 78.0 | 74.3 | 82.5 | 74.9 | 7.4 | 81.4 |

^a^ Rarified number of species based on 2,500 records per group shown in parentheses.

^b^ The four non-parametric estimators are based on species incidence (presence/absence): average-based coverage estimator (ACE), incidence-based coverage estimator (ICE), Chao2, and Jackknife 1. The mean (±SD) of the four estimators, and the percentage of species recorded (observed species/mean of estimator x 100) are also shown as a measure of sampling completeness (%).

Note: To prepare this table, we pooled scan records with data from 5-min focal-animal samples conducted during the intervals of scan samples during feeding bouts. Focal observations allowed recording rarely consumed species (*e.g.,* canopy climbers exploited during very short feeding bouts).
