# Supplementary material for: Feeding Strategies of Brown Howler Monkeys in Response to Variations in Food Availability
Source: PLoS One. 2016 Feb 5;11(2):e0145819. doi: 10.1371/journal.pone.0145819 (PMC4743924; doi:10.1371/journal.pone.0145819)
Supplement: S6 Table — (DOCX) [file pone.0145819.s009.docx]

**S6 Table. Relative and absolute density of adult individuals of the palm *Syagrus romanzoffiana* in each study site.**

| Study site | Fragment area (ha) | Relative density (inds./ha) | Absolute density (total number of inds.) |
| --- | --- | --- | --- |
| S1 | 1.6 | 0.6 | 1 |
| S2 | 9.5 | 0.3 | 3 |
| S3 | 2.9 | 0.3 | 1 |
| L1 | 93 | 0.7 | 65 |
| L2 | 106 | 0.3 | 32 |
| L3 | 108 | 0.2 | 22 |
